# Screening and identification of BP100 peptide conjugates active against *Xylella fastidiosa* using a viability-qPCR method

**DOI:** 10.1186/s12866-020-01915-3

**Published:** 2020-07-29

**Authors:** Aina Baró, Esther Badosa, Laura Montesinos, Lidia Feliu, Marta Planas, Emilio Montesinos, Anna Bonaterra

**Affiliations:** 1 Laboratory of Plant Pathology, Institute of Food and Agricultural Technology-CIDSAV-XaRTA, University of Girona, Girona, Spain; 2 LIPPSO, Department of Chemistry, University of Girona, Girona, Spain

**Keywords:** Antimicrobial peptides, *Xylella fastidiosa*, Viability-qPCR, PEMAX, Plant pathogens

## Abstract

**Background:**

*Xylella fastidiosa* is one of the most harmful bacterial plant pathogens worldwide, causing a variety of diseases, with huge economic impact to agriculture and environment. Although it has been extensively studied, there are no therapeutic solutions to suppress disease development in infected plants. In this context, antimicrobial peptides represent promising alternatives to traditional compounds due to their activity against a wide range of plant pathogens, their low cytotoxicity, their mode of action that make resistance more difficult and their availability for being expressed in plants.

**Results:**

Peptide conjugates derived from the lead peptide **BP100** and fragments of cecropin, magainin or melittin were selected and tested against the plant pathogenic bacteria *X. fastidiosa*. In order to screen the activity of these antimicrobials, and due to the fastidious nature of the pathogen, a methodology consisting of a contact test coupled with the viability-quantitative PCR (v-qPCR) method was developed. The nucleic acid-binding dye PEMAX was used to selectively quantify viable cells by v-qPCR. In addition, the primer set XF16S-3 amplifying a 279 bp fragment was selected as the most suitable for v-qPCR. The performance of the method was assessed by comparing v-qPCR viable cells estimation with conventional qPCR and plate counting. When cells were treated with peptide conjugates derived from **BP100**, the observed differences between methods suggested that, in addition to cell death due to the lytic effect of the peptides, there was an induction of the viable but non-culturable state in cells. Notably, a contact test coupled to v-qPCR allowed fast and accurate screening of antimicrobial peptides, and led to the identification of new peptide conjugates active against *X. fastidiosa*.

**Conclusions:**

Antimicrobial peptides active against *X. fastidiosa* have been identified using an optimized methodology that quantifies viable cells without a cultivation stage, avoiding underestimation or false negative detection of the pathogen due to the viable but non-culturable state, and overestimation of the viable population observed using qPCR. These findings provide new alternative compounds for being tested *in planta* for the control of *X. fastidiosa*, and a methodology that enables the fast screening of a large amount of antimicrobials against this plant pathogenic bacterium.

## Background

*Xylella fastidiosa* (Xf) is a xylem-limited Gram-negative bacterium transmitted by insect vectors that causes economically important plant diseases. Pierce’s disease of grapevine and Citrus Variegated Chlorosis were the most important diseases caused by Xf worldwide for many years [1, 2]. However, Xf recently emerged as a potential threat to European agriculture [3]. The outbreak of Xf in 2013 in Apulia (Italy) in oleander, almond and olive trees [4], and the detections in Corsica and Provence Alpes-Côte d’Azur (France), Alicante and the Balearic Islands (Spain), Tuscany (Italy), and Vila Nova de Gaia (Portugal) [5, 6] constitute an important change to its geographical distribution and adds new host plants.

The measures adopted in Europe are eradication of the infected plants to reduce inoculum sources to prevent the spread of the bacterium, the use of insecticides to control the vector population, and the use of pathogen-free plant material. However, these methods have been only partially successful and different strategies are being explored in order to find alternatives to achieve the management of diseases caused by Xf [7]. Direct strategies to control disease in affected hosts, based on chemical compounds like antibiotics, copper compounds, or biofilm inhibitors, either applied by sprays, drench or endotherapy, have failed to cure infected trees [8]. Therefore, there is a need for new and safe compounds for Xf disease management. Among the new compounds, antimicrobial peptides (AMPs) could be considered good candidates because they display activity against a wide range of plant pathogens, exhibit low cytotoxicity and their mode of action make more difficult the development of resistance [9–12]. In particular, a few AMPs with bactericidal activity against Xf have been reported, including cecropin A and B, magainin I and II, Shiva-1, indolicidin, PGQ, dermaseptin and gomesin [13–15]. Most of these peptides cause disruption of the cytoplasmic membrane, but also some of them have been described to interact with intracellular targets causing the inhibition of key processes [16]. Within our search for new AMPs to control plant diseases, we reported peptide conjugates incorporating units of the lead peptide **BP100** and fragments of cecropin A, magainin II or melittin, which were specifically designed to be expressed in plants [17, 18]. In fact, the peptide conjugate **BP178** was successfully expressed in rice endosperm, showing resistance against some plant pathogens [19]. This demonstrates the availability of these peptides for being produced by the plant itself, which could overcome the difficulties in accessing the vascular location of Xf observed by other treatment strategies. This family of peptides exhibited high antibacterial activity in vitro against plant pathogenic bacteria such as *Xanthomonas axonopodis* pv. *vesicatoria*, *Pseudomonas syringae* pv. *syringae* and *Erwinia amylovora*, were low haemolytic, and were able to control infections in plant hosts caused by these bacteria or even due to phytoplasms [10, 17, 20]. In the case of Xf, only **BP178** has been tested in vitro, showing high antibacterial activity against a collection of Xf strains. Its lytic activity upon Xf cells was identified as the main mode of action, with pore formation and disorganization of the cell membrane [21]. Therefore, we envisaged that this biological activity profile makes peptide conjugates derived from **BP100** good candidates to be tested against Xf. Since any sequence modification may influence their antimicrobial activity against Xf, as well as their stability and toxicity, a wide range of peptides must be screened to obtain the best candidates to be tested in plants.

Currently, there is a need for rapid, reliable and efficient methods useful for the screening of antimicrobial compounds against Xf due to the difficulties of culture of most of the strains and their slow growth [22]. Conventional methods, such as disk-diffusion test, broth or agar dilution assays, as well as antimicrobial gradient and automated instrument systems, rely on measuring growth inhibition using culture-based methods that are time consuming and unreliable for Xf [23]. Moreover, these methods may overestimate the antimicrobial activity of the tested compounds against Xf considering that its cells can enter in a viable-but-non-culturable state (VBNC) in response to harsh environments [24, 25]. Several methods have already been proposed to analyze only viable cells, such as ATP bioluminescence [23], direct microscopy or flow cytometry such as LIVE/DEAD® *Bac*light™ [26], DAPI combined with SYTOX Green [27], or 5-cyano-2,3-ditolyl tetrazolium chloride (CTC) that evaluates respiratory activity [28]. However, these methods are not able to specifically quantify viable target cells in mixture cultures. Alternative non culture-based methods would be more suitable to evaluate the efficacy of new compounds to inhibit Xf. Nucleic acid-based techniques such as quantitative PCR (qPCR) are commonly used to quantify total specific bacteria, as they can specifically detect target cells. All methods mentioned require specific sample preparation, training, and equipment. Nevertheless qPCR is particularly popular because it has been used for a wide number of applications and has become a standard equipment in researcher laboratories, so methods that use qPCR are easier to be performed anywhere.

A limitation of the qPCR is the overestimation of alive cells. Due to the fact that DNA can persist for an extended period after cell death [29], the DNA of both viable and dead cells is amplified. In contrast, the viable quantitative PCR (v-qPCR) allows the quantification of only viable cells. Generally, v-qPCR uses the nucleic acid-binding dyes propidium monoazide (PMA or PMAxx) or ethidium monoazide (EMA) in combination with qPCR for selectively detecting and enumerating viable cells. Both PMA and EMA bind to the free DNA and the DNA of dead cells with damaged membranes. In addition, EMA binds to the DNA of non-metabolically active cells with an intact membrane, avoiding its subsequent amplification by qPCR. In the PEMAX reagent, an optimized mixture of PMA (≥20 μM) and EMA (< 10 μM) is used [30]. This low level of EMA is accumulated inside non-metabolically active cells that still have an intact cell membrane, while it is eliminated from viable cells through active transport. Therefore, after treatment with PEMAX, only the DNA of viable cells remains unlabelled and is detected by qPCR [31, 32]. This methodology has already been used for foodborne pathogenic bacteria in different matrices [33], to monitor biological control agents in field studies [34] and, in the case of Xf, to differentiate viable cells under stressing conditions [35, 36]. Nevertheless, v-qPCR using the PEMAX reagent has never been optimized as a screening methodology for the identification of antimicrobials active against Xf.

For the development of a v-qPCR assay for the detection and quantification of Xf it is necessary to find a molecular marker species-specific suitable to be used with PEMAX. Different primer pairs and probes specific for Xf detection have been described and validated [37–40]. Primer pairs normally show different amplification efficiencies and levels of sensitivity depending on the target site, the nature of the primers and the length of the amplicon. Moreover, suppression of dead cells amplification after PEMAX treatment is also dependent on the length of the DNA fragment amplified by qPCR, as the probability of dye binding increases in longer target regions [34].

The aim of the present work was to find peptide conjugates derived from **BP100** highly active against Xf in vitro*.* To accomplish this purpose, firstly a screening methodology based on a contact test combined with a v-qPCR method was optimized for representative strains of Xf and for an accurate and reliable evaluation of the antimicrobial activity of peptides. Afterwards, a set of peptide conjugates derived from **BP100**, designed for being expressed in plant systems and active against other plant pathogens, were selected and screened using the optimized methodology to evaluate its antimicrobial activity against Xf.

## Results

### Amplification efficiency and sensitivity of qPCR assays

Eight TaqMan based qPCR assays amplifying three different gene sequence targets of Xf and producing different amplicon lengths were checked in order to study their suitability for v-qPCR (Table 1). Standard curves of the eight qPCR assays showed good linearity over 7-log range, from 1 × 10^2^ to 1 × 10^8^ CFU/ml, reporting R^2^ values over 0.99 (Additional file 1). Table 1 shows the amplification efficiency and the sensitivity of each qPCR assay. All amplification efficiencies were higher than 94%, and did not vary between qPCR assays having the same target gene, except for the Elongation factor Tu (EFTu), which ranged from 95 to 98%. The three qPCR assays amplifying part of the 16S rRNA gene (XF16S) displayed the best amplification efficiencies (97%).

**Table 1:** Primers and TaqMan probes used for qPCR analysis, amplification efficiency and sensitivity analysis

qPCR assay	Primer/ probe	Sequence	Amplicon length (bp)	Slope	R^2^	Efficiency (%)	Sensitivity^a^	Reference of source
HL-1	rev-2	GGTTTTGCTGACTGGCAACA	221	−3.47	0.99	94	30.8	37
HL-2	rev-3	CACTTGTGGTAAGCATCCTGAG	307	−3.49	0.99	94	31.8	This study
	for	AAGGCAATAAACGCGCACTA						37
	probe	FAM/−TGGCAGGCAGCAACGATACGGCT−/BHQ						37
XF16S-1	rev-1	CCGATGTATTCCTCACCCGTC	62	−3.39	0.99	97	27.2	39
XF16S-2	rev-2	CTAATCGGACATCGGCTCAT	181	−3.39	0.99	97	28.1	This study
XF16S-3	rev-3	GTAGGAGTCTGGACCGTGTCTC	279	−3.39	0.99	97	29.7	21
	for	CGGCAGCACGTTGGTAGTAA						39
	probe	FAM/−CATGGGTGGCGAGTGGC−/TAMRA						39
EFTu-1	rev-1	GGCGAGCCAACAAAATGTGTT	77	−3.21	0.99	95	28.4	38
EFTu-2	rev-2	ATCACCAGGAAAATCATACTTGCT	202	−3.38	0.99	98	29.4	This study
EFTu-3	rev-3	GAATGTGGGTATCCAATGCTTC	311	−3.21	0.99	95	33.9	This study
	for	GGATGGTGCGATTTTAGTATGTTCT						38
	probe	FAM/−TGATGGTCCGATGCCTCAGACTCGT−/TAMRA						38

^a^*C*_*T*_ value at a concentration of 5 × 10^3^ CFU/ml

Regarding the sensitivity, the eight qPCR assays were very different at 5 × 10^3^ CFU/ml cycle threshold values (*C*_*T*_), ranging from 27.2 to 33.9. Again, the three assays amplifying part of the XF16S gene displayed the higher sensitivity. In all cases, qPCR assays amplifying larger DNA fragments (311, 279, and 307 bp) were less sensitive than the ones generating shorter amplicons. The XF16S-3 design (279 bp) showed sensitivity values comparable to the ones obtained with the qPCR assays amplifying fragments of less than 100 bp. Due to the fact that higher amplicon lengths are more suitable when using PEMAX, the qPCR assay with XF16S-3 was selected for further experiments.

### V-qPCR

The effect of different PEMAX concentrations on the amplification of DNA targets of viable and dead Xf subsp. *fastidiosa* strain Temecula (Xff) cells was studied by determining the signal reduction value (SR), defined as the difference of *C*_*T*_ value between PEMAX and non-PEMAX treated samples (Δ*C*_*T*_) (Additional file 2). On viable cells, no significant differences on SR values were observed when using a PEMAX concentration of 2.5, 5, 7.5 and 50 μM. However, at 10 μM, the SR value was significantly higher compared to 5 and 7.5 μM. Regarding dead cells, significant differences were observed between PEMAX concentrations, being 7.5 and 10 μM the concentrations with highest SR values. Based on these results, a PEMAX concentration of 7.5 μM was chosen for further experiments, as it was the lowest concentration that allowed a better discrimination between viable and dead cells.

**Fig. 1:**
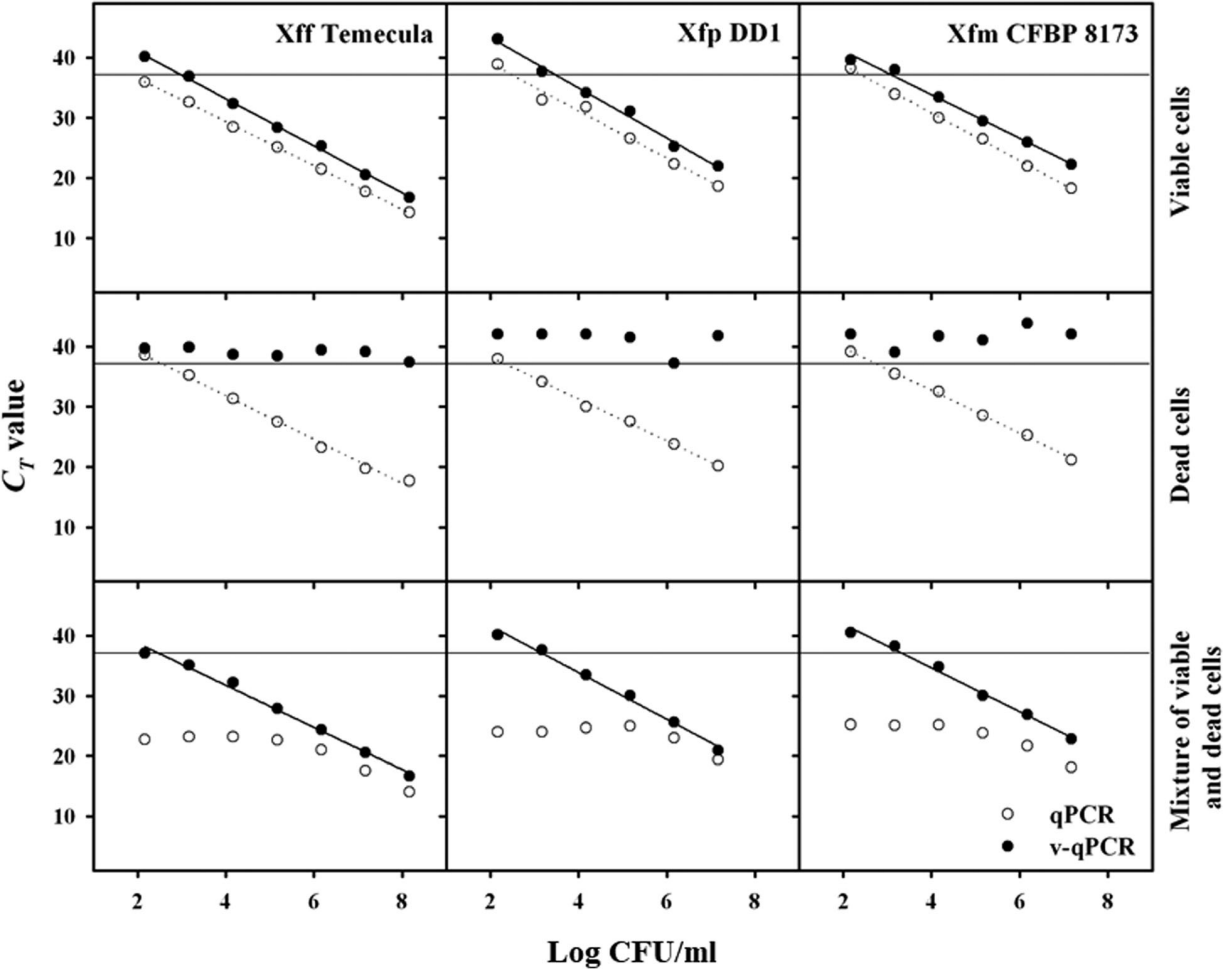
Relationship between *C*_*T*_ values and cell concentration in three strains of Xf using conventional qPCR (white symbols) and v-qPCR (black symbols), for viable cells, dead cells, and a mixture of viable cells with a fixed concentration of dead cells (1 × 10^6^ CFU/ml). TaqMan-based qPCR assay done with XF16S-3 primers. The thin line represents the detection limit at *C*_*T*_ = 37.5

Standard curves were performed using cell suspensions of Xff, Xf subsp. *pauca* (Xfp) and Xf subsp. *multiplex* (Xfm) to evaluate the suitability of the v-qPCR method to quantify viable cells. PEMAX and non-PEMAX-treated standard curves showed good linearity between 1 × 10^2^ and 1 × 10^7^ CFU/ml, with R^2^ values above 0.985. In all cases, a shift of around 2 cycles was observed when comparing PEMAX treated and non-treated samples from the same subspecies (Fig. 1). This variation was already observed in the optimization of the PEMAX concentration (Additional file 2). Amplification efficiencies of all standard curves were around 80% and values were comparable among subspecies (88.1% without PEMAX and 80% with PEMAX for Xff, 83.2% without PEMAX and 77.1% with PEMAX for Xfp, and 79.5% without PEMAX and 80.8% with PEMAX for Xfm). In dead cells, samples ranging from 1 × 10^3^ to 1 × 10^7^ CFU/ml treated with PEMAX displayed *C*_*T*_ values higher than 37.5, indicating, as expected, an inhibition of their amplification (Fig. 1). In mixtures of viable cells (from 1 × 10^3^ to 1 × 10^7^ CFU/ml) and dead cells (fixed quantity of 1 × 10^6^ CFU/ml), standard curves showed a high correlation coefficient (R^2^ values above 0.99) when samples were treated with PEMAX (Fig. 1). Amplification efficiencies calculated were similar to the ones obtained in the standard curves of only viable cells (92.9% for Xff, 80.9% for Xfp and 80.8% for Xfm), indicating that presence of DNA from dead cells do not interfere in the amplification of DNA from viable cells.

### Antimicrobial activity of peptide conjugates derived from BP100: optimization of the contact test

To develop a method for screening antimicrobial activity of AMPs against Xf, different contact test conditions were studied, such as Xff cell concentration, contact test time and AMP concentration. Loss of viability after the contact test was assessed by v-qPCR and compared with plate counting (culturable cells) and qPCR (total cells).

**Fig. 2:**
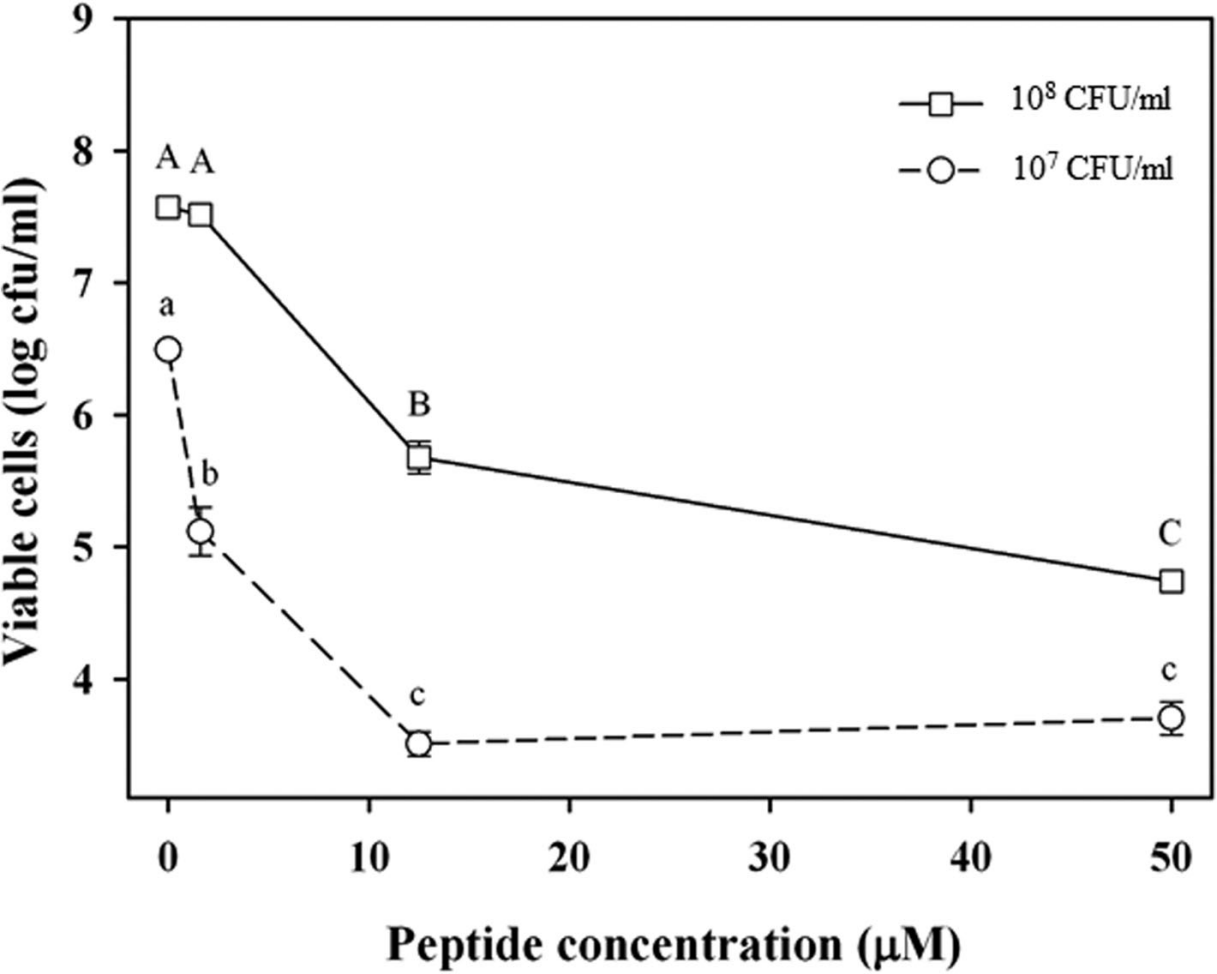
Effect of peptide **BP178** on viability of Xff strain Temecula estimated by v-qPCR at different peptide concentrations (1.6, 12.5 and 50 μM). Two assays were performed at different initial Xff cell concentrations, 1 × 10^7^ CFU/ml (circles) and 1 × 10^8^ CFU/ml (squares). The exposure time to the peptide was 3 h. Xff concentration in non-treated cells was estimated after 3 h by v-qPCR. The detection limit of the v-qPCR is 3 log CFU/ml. Values are the means of three replicates, and error bars represent the standard deviation of the mean. Lowercase letters correspond to the means comparison of viable cells in 1 × 10^7^ CFU/ml. Capital letters correspond to the means comparison of viable cells in 1 × 10^8^ CFU/ml. Means sharing the same letters are not significantly different (*P* < 0.05), according to the Tukey’s test

The antimicrobial activity of **BP178** at 1.6, 12.5 and 50 μM was studied against two different Xff cell concentrations in a 3 h contact test (Fig. 2). At all peptide concentrations, Xff cells showed higher loss of viability (expressed as log reduction of cell viability) at 1 × 10^7^ CFU/ml than at 1 × 10^8^ CFU/ml, indicating a significant effect of the initial cell concentration (*P* < 0.001). Specifically, treatment of Xff cells at 1 × 10^7^ CFU/ml with **BP178** at 1.6 μM caused a significant reduction of viable cells (1.5 log), while no significant reduction was observed at 1 × 10^8^ CFU/ml. At 12.5 μM, significant reduction of viable cells was observed in both cases, being 3 log reduction at 1 × 10^7^ CFU/ml whereas 2 log reduction at 1 × 10^8^ CFU/ml. A peptide concentration of 50 μM, both Xff cell concentrations exhibited a similar reduction of viability of around 3 log.

**Fig. 3:**
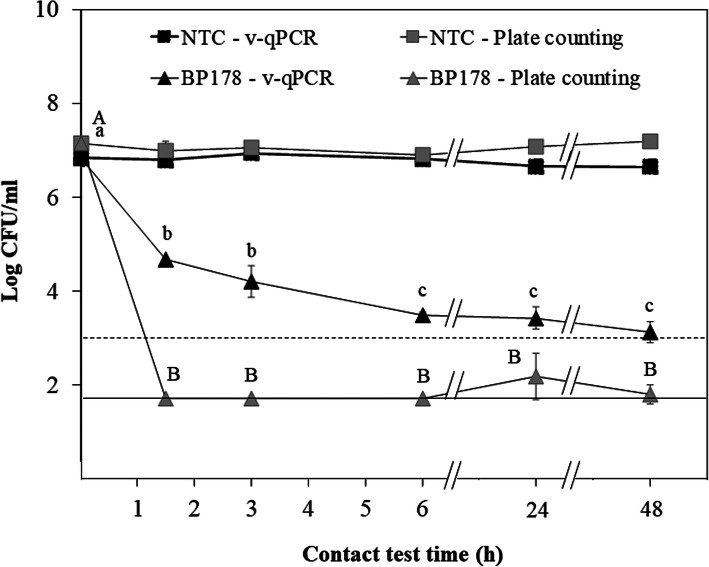
Effect of peptide **BP178** on viability and culturability of Xff strain Temecula at different exposure times. Cell viability was estimated by v-qPCR (black symbols) and cell culturability by plate counting (grey symbols). Initial cell concentration was 1 × 10^7^ CFU/ml and the **BP178** concentration used was 50 μM. Non-treated controls (NTC) were also performed by adding the corresponding volume of sterile distilled water. The dash line represents the detection limit of v-qPCR, whereas the normal line indicates the detection limit of the plate counting technic. Values are the means of three replicates, and error bars represent the standard deviation of the mean. Lowercase letters correspond to the means comparison of viable cells treated with **BP178** (black triangles). Capital letters correspond to the means comparison of culturable cells treated with **BP178** (grey triangles). Means sharing the same letters are not significantly different (*P* < 0.05), according to the Tukey’s test

The effect of peptide **BP178** on viability and culturability at different contact test times (from 1.5 to 48 h) was studied (Fig. 3). **BP178** at 50 μM reduced (*P* < 0.001) viable and culturable cells of Xff in all exposure times. There were significant differences (*P* < 0.001) between v-qPCR (viable cells) and plate counting (culturable cells) in Xff suspensions mixed with **BP178**. In the case of v-qPCR, a progressive viability reduction occurred up to a contact test time of 6 h (between 2 and 3.5 log reduction), practically reaching the detection limit of the method (3 log CFU/ml). In contrast, the culturability of the cells mixed with the peptide dropped abruptly to levels near the detection limit (1.5 log CFU/ml) after 1 h of incubation. Xff cells maintained similar levels of both, cell viability (v-qPCR) and cell culturability, in the non-treated control (without **BP178**) over 48 h.

**Fig. 4:**
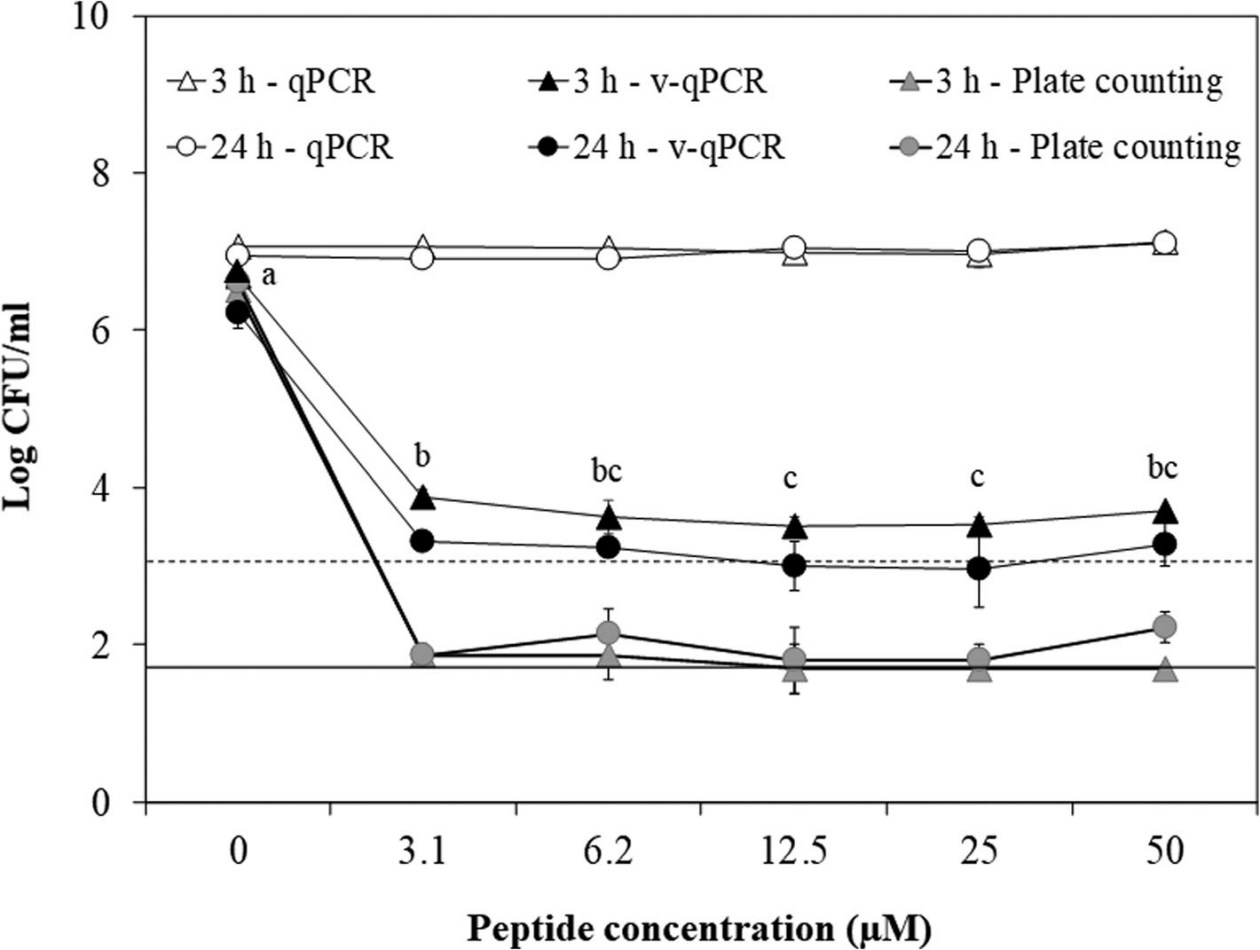
Effect of peptide **BP178** on viability and culturability of Xff strain Temecula at different peptide concentrations. Total cell concentration was estimated by conventional qPCR (white symbols), cell viability was estimated by v-qPCR (black symbols), and cell culturability by plate counting (grey symbols). Exposure times of 3 h (triangles) and 24 h (circles) were used. Cell concentration was 1 × 10^7^ CFU/ml in both cases. The dash line represents the detection limit of v-qPCR, whereas the normal line indicates the detection limit of the plate counting technic. Values are the means of three replicates, and error bars represent the standard deviation of the mean. Letters correspond to the means comparison of viable cells treated with **BP178** at exposure time of 3 h. Means sharing the same letters are not significantly different (*P* < 0.05), according to the Tukey’s test

The AMP concentration was evaluated by assessing the loss of viability of Xff suspensions mixed with **BP178** at 3.1, 6.2, 12.5, 25 and 50 μM at a contact time of 3 and 24 h (Fig. 4). In this experiment, there were also significant differences (*P* < 0.001) between v-qPCR (viable cells), plate counting (culturable cells) and qPCR (total cells) in Xff suspensions in the presence of different concentrations of **BP178**. In the case of v-qPCR, a similar reduction of cell viability was observed (around 3 log reduction) for all **BP178** concentrations in both contact test times (3 and 24 h), and only differences between the incubation periods were observed at 3.1 μM and 50 μM (*P* < 0.001). Comparing peptide concentrations, a progressive viability reduction occurred in the contact test of 3 h and significant differences were observed between 0, 3.1 and 12.5 μM. The culturability of Xff cells was also reduced without significant differences in almost all peptide concentrations at both incubation periods (around 5 log cell culturability reduction). The minimal bactericidal concentration (MBC) of **BP178** was determined to be 3.1–6.25 μM, which corresponds to 10–20 μg/ml.

### Screening of peptide conjugates derived from BP100 against Xff

Eleven selected conjugates derived from **BP100** were tested against Xff at 3.1 and 12.5 μM (Table 2). Peptide **tag54**, an epitope tag designed for being used in peptide detection and purification, was included as a control to check the effect upon Xff cells of a peptide that previously showed no antimicrobial activity against other plant pathogenic bacteria [17]. **BP100** and **BP178** were also assayed for comparison purposes. Peptide **tag54** did not show antimicrobial activity against Xff cells. **BP100** led to a log reduction (N_0_/N) of cell viability of 1.39 at 3.1 μM and of 3.27 at 12.5 μM. At 3.1 μM, all peptide conjugates showed antimicrobial activity with a log reduction of cell viability between 0.91 and 2.95. At 12.5 μM, a higher effect was observed for all peptides, leading to an Xff viability reduction between 1.33 and 3.79 log. **BP171**, **BP175** and **BP178** were highly active, with a 3.5–4 log reduction of cell viability at 12.5 μM of peptide concentration and 2.5–3 log reduction at 3.1 μM. **BP170**, **BP176** and **BP180** were moderately active, with a 3–3.5 log reduction at 12.5 μM and 2–3 log reduction at 3.1 μM. **BP181**, **BP188**, **BP192**, **BP198** and **BP213** were low active, with less than 3 log reduction at 12.5 μM and less than 2 log reduction at 3.1 μM. Highly and moderately active peptides have a significantly different log reduction compared to low active peptides (according to the mean separation test). The highly active peptides were conjugates incorporating a **BP100** unit and a melittin or a magainin fragment. In particular, **BP171**, containing **BP100** and a melittin fragment, led to a log reduction of 3.79 at 12.5 μM and of 2.91 at 3.1 μM, while **BP175** and **BP178,** which incorporate **BP100** and a magainin fragment, led to a log reduction of 3.52 and 3.54 at 12.5 μM, and of 2.65 and 2.95 at 3.1 μM, respectively.

**Table 2:** Screening of conjugate peptides derived from **BP100** against Xff, compared with **BP100** and **tag54,** by means of a contact test combined with v-qPCR method

Peptide type	Code	Sequence	N° of AA	Log N_0_/N^a^			
				3.1 μM		12.5 μM	
Reference peptides	**tag54**	KDWEHLKDWEHLKDWEHL-OH	18	0 ± 0	g	0 ± 0	G
	**BP100**	KKLFKKILKYL-NH_2_	11	1.39 ± 0.07	d	3.27 ± 0.09	ABC
**BP100** (dimer)	**BP192**	KKLFKKILKYL - **AGPA** - KKLFKKILKYL - **KDEL**-OH	30	0.91 ± 0.13	e	1.33 ± 0.11	F
	**BP198**	KKLFKKILKYL - KKLFKKILKYL - **KDEL**-OH	26	1.37 ± 0.04	d	1.94 ± 0.08	DE
	**BP213**	KKLFKKILKYL - **AGPA** - LYKLIKKFLKK - **KDEL** - OH	30	1.17 ± 0.02	de	1.34 ± 0.10	F
**BP100** - Melittin [10–19]	**BP170**	KKLFKKILKYL - TTGLPALISW - OH	21	2.89 ± 0.09	ab	3.11 ± 0.06	BC
	**BP171**	KKLFKKILKYL - **AGPA** - TTGLPALISW-OH	25	2.91 ± 0.02	ab	3.79 ± 0.15	A
**BP100** - Magainin [4–10]	**BP180**	KKLFKKILKYL - KFLHSAK-OH	18	2.29 ± 0.27	c	2.96 ± 0.19	C
	**BP181**	KKLFKKILKYL - **AGPA** - KFLHSAK-OH	22	1.30 ± 0.08	de	2.40 ± 0.37	D
**BP100** - Magainin [1–10]	**BP175**	KKLFKKILKYL - **AGPA** - GIGKFLHSAK-OH	25	2.65 ± 0.18	abc	3.52 ± 0.16	AB
	**BP176**	KKLFKKILKYL - GIGKFLHSAK-OH	21	2.47 ± 0.12	bc	3.34 ± 0.11	ABC
	**BP178**	KKLFKKILKYL - **AGPA** - GIGKFLHSAK - **KDEL**-OH	29	2.95 ± 0.22	a	3.54 ± 0.05	AB
**BP100 -** Cecropin A [25–37]	**BP188**	KKLFKKILKYL - AVAVVGQATQIAK - **KDEL**-OH	28	0.89 ± 0.04	e	1.60 ± 0.09	EF

^a^Log reduction of Xff cell viability after the treatment with the peptides (at 3.1 and 12.5 μM) for 3 h was calculated as log N_0_/N, where N_0_ is the initial number of viable cells and N is the number of viable cells after the treatment, as estimated by v-qPCR. Values are the mean of three replicates, the confidence intervals are indicated. Lowercase letters correspond to the means comparison of Log N_0_/N in 3.1 μM. Capital letters correspond to the means comparison of Log N_0_/N in 12.5 μM. Means sharing the same letter indicate no significant differences between peptides (*P* < 0.05), according to the Tukey’s test

The antibacterial activity of **BP171** and **BP198** was also evaluated at different peptide concentrations by v-qPCR and plate counting (Additional file 3). Results showed that, as expected, both methods classified **BP171** as highly active against Xff and **BP198** as a low active peptide against this pathogen. After **BP171** treatment, the Xff viable and culturable cells reached the detection limit in both technics, whereas **BP198** was not able to completely inactivate Xff, neither using plate counting nor v-qPCR.

## Discussion

The difficulties in managing diseases caused by Xf have stimulated the search for novel bactericides. Several antimicrobial compounds, such as toxins, antibiotics, phenolic acids and AMPs, have been reported to be active against several Xf strains with MBC or minimal inhibitory concentrations (MIC) ranging from 8 to 800 μM [14, 15, 41–44]. Interestingly, the AMPs magainin I and II, and dermaseptin have been reported to display low MIC or MBC values against Xf [14]. In addition, AMPs such as the lytic peptides LIMA-A and cecropin B have been expressed in grapevines resulting in a successful control of Xf in greenhouse conditions [45, 46]. So, their antibacterial activity and their availability for being expressed in plants make AMPs good candidates for the control of this plant pathogen, either using transgenic expression or other delivery strategies such as endotherapy. In the present study, a set of 11 peptide conjugates derived from the lead peptide **BP100** and a fragment of cecropin, magainin or melittin, previously reported by our group as active against several plant pathogenic bacteria and with low toxicity to eukaryotic cells (moderate to low hemolysis) (Table 3) [10, 17, 20], were screened for their activity against Xf. One of these peptide conjugates (**BP178**) has been produced in transgenic rice [18, 19], and has also been tested in vitro against Xf and other plant pathogens showing high antibacterial activity [17, 21].

**Table 3:** MIC against different plant pathogens and hemolysis percentage displayed by the peptide conjugates derived from **BP100** tested in this study

Peptide type	Code	MIC (μM)			Hemolysis^**d**^ (%)		
		***Xav***^**a**^	***Psa***^**b**^	***Ea***^**c**^	50 μM	150 μM	250 μM
Reference peptides	**tag54**	> 100	> 100	> 100	0	0	1
	**BP100**	10–20	7.5–10	7.5–10	1	8	18
**BP100** (dimer)	**BP192**	7.5–10	7.5–10	7.5–10	51	69	69
	**BP198**	10–20	10–20	10–20	59	72	72
	**BP213**	1.25–2.5	2.5–5.0	2.5–5.0	90	92	98
**BP100** - Melittin [10–19]	**BP170**	1.25–2.5	2.5–5.0	2.5–5.0	82	93	98
	**BP171**	2.5–5.0	1.25–2.5	2.5–5.0	5	16	34
**BP100** - Magainin [4–10]	**BP180**	2.5–5.0	2.5–5.0	2.5–5.0	12	54	58
	**BP181**	2.5–5.0	1.25–2.5	2.5–5.0	0	0	0
**BP100** - Magainin [1–10]	**BP175**	1.25–2.5	2.5–5.0	5.0–7.5	6	14	32
	**BP176**	2.5–5.0	2.5–5.0	5.0–7.5	3	44	59
	**BP178**	2.5–5.0	2.5–5.0	2.5–5.0	0	3	25
**BP100 -** Cecropin A [25–37]	**BP188**	2.5–5.0	1.25–2.5	2.5–5.0	10	24	42

^a^*Xav*, *Xanthomonas axonopodis* pv. vesicatoria; ^b^*Psa*, *Pseudomonas syringae* pv. syringae; ^c^*Ea, Erwinia amylovora*; ^d^Percent hemolysis plus confidence interval (α = 0.05)

A methodology consisting of a contact test combined with a v-qPCR method was developed in the present work in order to screen the activity of AMPs against the fastidious bacterium Xf. The v-qPCR method has the advantage to allow the quantification of viable cells, including VBNC and culturable cells, without a cultivation stage. Other studies used a variety of culture-dependent methods to evaluate the antimicrobial activity against Xf. Nevertheless, for the screening of large amounts of antimicrobial peptides, these methodologies are time consuming, as they require incubation periods of several days for Xf to grow. While the v-qPCR can be performed in less than 1 day, about 4–7 days are required for the agar plate dilution assay, for the contact test followed by plate counting or for the agar disc diffusion method [14, 15, 42, 44].

The v-qPCR has been efficiently used for the monitoring of microorganisms with biotechnological potential [31], and for the detection and quantification of human pathogens in food [47] or in the environment [48]. In particular, in the case of Xf, different PCR assays are commonly used for the detection and quantification, and v-qPCR methods in combination with EMA or PMAxx reagents were also reported to discriminate between viable and membrane-damaged cells [35, 36]. In our work the PEMAX reagent, an optimized mixture of EMA and PMA that has been previously proven to be efficient in discriminating viable from dead cells in a biological control agent was used [34]. The effect of PEMAX concentration was optimized in order to detect only viable Xf cells. PEMAX at 7.5 μM was the lowest concentration showing good results as inhibited the DNA amplification of dead Xff cells at 1 × 10^7^ CFU/ml while viable cells were not affected. Lower concentrations, 2.5 and 5 μM, were less effective in preventing DNA amplification of high cell concentrations, probably due to the lack of available reagent. In contrast, higher concentrations of PEMAX, 10 and 50 μM, caused a slight toxicity effect on Xff cells. In other studies, a PEMAX concentration of 50 μM has been reported to be the optimal to detect *Lactobacillus* and *Salmonella* using a v-qPCR assay [31, 34]. However, it has also been described that excessive concentrations of these two dyes causes toxicity in some microorganisms [36, 49]. Therefore, the concentration of PEMAX has to be optimized for each species in order to allow DNA amplification of only viable cells, without being toxic to the bacteria.

In order to choose the best conditions for v-qPCR, eight Xf-specific qPCR assays with different amplification sites and lengths were compared. All assays showed acceptable and similar efficiency percentages that were in agreement with those observed in other qPCR designs used for the quantification of Xf [50, 51]. In contrast, the assays differed in the sensitivity values. The primer pair (XF16S-3), chosen for further assays with a length of 279 bp, exhibited sensitivity values similar to those previously reported [50, 51]. As it has been described [34, 52], the amplicon length is an important parameter to consider when optimizing a v-qPCR because there is a higher probability of dye intercalation in cell-free DNA when using long length amplicons compared to short length amplicons. The reliability of the v-qPCR when using XF16S-3 as primer pair and a PEMAX concentration of 7.5 μM was evaluated and validated on viable and dead cells, and on a mixture of viable and dead cells of Xff, Xfp and Xfm. v-qPCR method developed showed acceptable amplification efficiencies and correlation coefficient values. Although both, the use of longer amplicons and the presence of PEMAX, decreased the sensitivity of the qPCR, a *C*_*T*_ value corresponding to 1 × 10^3^ CFU/ml viable cells was determined as the detection limit of the developed v-qPCR.

To set up the conditions of the contact test, the initial cell concentration of Xff, the contact test time and the peptide concentration were optimized. Considering other studies, which employed Xf cell concentrations ranging from 1 × 10^5^ to 1 × 10^8^ CFU/ml to test antimicrobial compounds [14, 41, 42], a Xff concentration of 1 × 10^7^ CFU/ml was chosen as it brought out the effect of the AMP at low concentration and enabled a viability reduction of 4 log before reaching the detection limit of the v-qPCR method (1 × 10^3^ CFU/ml). At 1 × 10^8^ CFU/ml, a different viability reduction pattern was observed. As described, antimicrobial peptides (and other antimicrobials) are quenched during interaction with target cells due to their binding to the cell through time. Because there is a threshold number of peptide molecules necessary to kill a target cell, the viability reduction is not only dependent on the antimicrobial concentration but also on the target bacteria concentration [53, 54]. Regarding the contact test time, a lethality percentage around 99.8% was observed after 3 h of contact test with the peptide. Against Xf, a contact test time of 18 h has been employed [15] but as reported, the bactericidal effect of an antimicrobial compound is time-dependent and a lethality percentage of 90% after 6 h is equivalent to a 99.9% of dead cells after 24 h [23]. Therefore, in our study a contact test of 3 h allows fast screening of AMPs against Xf with similar results than longer contact test times. Finally, peptide concentrations of 3.1 and 12.5 μM were the ones selected for the screening of AMPs because it was envisaged that they would allow the classification of the peptides according to their activity against Xf.

The suitability of the v-qPCR method to estimate the viability of Xf cells after the contact test was studied by comparing it with qPCR and plate counting onto PD2 agar plates. No significant differences were observed between the three methods in untreated Xff cells. However, in cells treated with the peptide conjugates derived from **BP100**, qPCR overestimated viable cells (around 4 log units) compared to v-qPCR, indicating the presence of DNA from dead cells, and plate counting underestimated the viability of Xff (around 2 log units). While previous research has focused on determining the activity of antimicrobial compounds using methodologies that report information about the culturable cells [14, 15, 41, 42], v-qPCR offers the possibility of determining the amount of viable cells, irrespective of their culturability. In the present study, it was observed that viability of Xf cells was progressively reduced after the treatment with AMPs, while culturability dropped abruptly to levels near the detection limit. This fact is probably due to the formation of metabolically active persistent cells (VBNC state). It has been described that Xf cells enter in the VBNC state when they are exposed to inhibitory concentrations of antimicrobial compounds [25, 35, 55, 56]. In other plant pathogens, VBNC cells have been reported to have the capacity to revert its physiological state and acquire again its virulence, being widely responsible for recalcitrant infections [57, 58]. Taking this into account, the quantification of the whole viable fraction (including VBNC and culturable cells) is necessary to determine the antimicrobial activity of compounds because the presence of these cells can play a significant role in terms of defining their pathogenicity and epidemiology.

Remarkably, the use of the above described contact test coupled with the v-qPCR for the screening of peptides allowed a rapid and reliable identification of sequences among the peptide conjugates derived from **BP100** active against Xf, and their classification as: (i) highly active (**BP171**, **BP175**, **BP178**), (ii) mid active (**BP170**, **BP176**, **BP180**), and (iii) low active (**BP181**, **BP188**, **BP192**, **BP198**, **BP213**). The best peptides **BP171**, which incorporates **BP100** and a melittin fragment, and **BP175** and **BP178**, which result from the conjugation of **BP100** with a magainin II fragment, showed higher activity than **BP100**. Other AMPs, such as gomesin, dermaseptin or magainin II, have been reported to be active against Xf (MIC or MBC of 4.5–9, 8–32 and 8–64 μg/ml, respectively) [14, 15]. Unfortunately, it is not possible to compare these activity values obtained using v-qPCR because the methods used in other works to assay the antibacterial activity were different. However, the activity values of **BP171** and **BP178** determined in our work using plate counting, which attained a MBC between 1.5 and 3.1 μM (~ 5–10 μg/ml) and 3.1 and 6.1 μM (~ 10–20 μg/ml) respectively, can be compared and are similar to the gomesin values, since the method used in both cases was a contact test followed by plate counting.

## Conclusions

This work has allowed the fast screening and identification of five new bactericidal peptide conjugates (**BP171**, **BP175**, **BP170**, **BP176**, **BP180**) active against Xf, in addition to the previously described **BP178**. All of them can be considered as candidates for the development of new agents to treat the plant diseases caused by this bacterium. The contact test combined with v-qPCR method has the advantage of quantifying only viable Xf cells, therefore the evaluation of the antimicrobial effect of AMPs is more precise. Moreover, considering the European Union rules for quarantine organisms, and particularly for Xf, the method minimizes the risk of dissemination of the pathogen, as it allows working in more safe conditions (shorter periods of time with manipulating living cells), compared to the culture-based methods. Apart from testing AMPs and other antimicrobials against Xf in vitro, the method could also be used in plants, as the Xf population quantified in naturally infected olive trees and in artificially inoculated grapevines is around 10^7^–10^8^ CFU/ml [36, 59]. This would be of interest to confirm the antimicrobial activity of the AMPs against the pathogen in their hosts. In addition, the fact that these conjugates were designed to be expressed in plants extends their possible technological use by means of transgenic plant hosts producing peptides to kill the pathogen [45, 46, 60, 61].

## Methods

### Xf strains, growth conditions and DNA extraction

Xff strain Temecula 1 ATCC 700964 [62], Xfp strain DD1 [63] and Xfm strain CFBP 8173 [64] were used. All strains were grown in BCYE agar [65] at 28 °C for 1 week and were stored in PD2 broth [66] with 30% glycerol at − 80 °C. Cell suspensions were prepared in sterile succinate-citrate-phosphate (SCP) buffer [40] at 1 × 10^8^ CFU/ml (optical density at 600 nm being 0.3, confirmed by colony counts) and diluted to appropriate concentrations. DNA was extracted using GeneJET Genomic DNA Purification Kit (Thermo Fisher Scientific, Waltham, USA) following the specific protocol for Gram-negative bacterial suspensions. Briefly, 200 μl were centrifuged at 15,900 x g during 10 min, the pellet was resuspended in 180 μl of digestion solution and 20 μl of proteinase K. Samples were incubated at 56 °C for 30 min, then 20 μl of RNase solution was added and another incubation step of 10 min at room temperature was carried out. Next, 200 μl of lysis solution were added, followed by 400 μl of 50% ethanol, and all the volume was transferred to a GeneJET Genomic DNA Purification Column. Two washes were performed using two different wash buffers, and finally DNA was re-suspended with 30 μl of PCR-grade water. DNA was stored at − 20 °C for further analysis.

### qPCR design: evaluation of the amplification efficiency and sensitivity

qPCR assays were conducted using the primer pairs and TaqMan probe sets described in Table 1. Primer3Plus software was used to obtain amplicons with different length that shared the described forward primers and probes but with new different reverse primers. All qPCR were performed using 96-well plates containing 12.5 μl 2X TaqMan Universal PCR Master Mix (Thermo Fisher Scientific, USA), final concentrations of 400 nM for each forward and reverse primer and of 150 nM for TaqMan probe with dye, 8.46 μl of PCR-grade water and 2 μl of template DNA in each well. Serial 10-fold dilutions of Xff covering a 7-log range (from 1 × 10^2^ to 1 × 10^8^ CFU/ml) were prepared in sterile SCP buffer and each concentration was performed in triplicate. DNA extraction from each suspension was performed as described above. All reactions were performed in duplicate and carried out in a QuantStudio 5 real-time PCR system (Applied Biosystems, Foster City, CA, USA). qPCR conditions were 95 °C for 10 min for enzyme activation followed by denaturation at 95 °C for 1 min, and extension and annealing at 59 °C for 1 min. The qPCR was run for 45 cycles. Standard curves were developed to check the sensitivity and efficiency of the qPCR assays. *C*_*T*_ values were plotted against the logarithm of the initial number of CFU/ml to determine the amplification efficiency of each design using the following equation.

$$ \mathbf{E}\left(\%\right)=\left({10}^{-1/ slope}-1\right)\times 100 $$

### V-qPCR: optimization of the PEMAX concentration

A stock solution of 2000 μM of PEMAX reagent (GenIUL, Terrassa, Spain) was prepared and stored as described [32]. To optimize the concentration of PEMAX, 20 μl of PEMAX stock solutions at 25, 50, 75, 100 or 500 μM were added into 180 μl of viable or dead Xff cell suspension, both adjusted to 1 × 10^7^ CFU/ml in SCP. Dead cells were obtained by heating the cell suspension at 95 °C for 10 min (ThermoMixer F1.5; Eppendorf, Hamburg, Germany), and the suspension was plated on PD2 agar and incubated for 1 week at 28 °C to check the absence of growth. PEMAX treated samples were thoroughly mixed and incubated for 30 min in the dark at room temperature with manual shaking every 10 min. Next, samples were photoactivated with the PhAST Blue photoactivation system (GenIUL, Barcelona, Spain) for 15 min with intensity of 100%. Each PEMAX treated sample was transferred into DNA low-binding 1.5 ml tube (Sarstedt, Nümbrecht, Germany) and collected by centrifugation at 15,900 x g for 10 min. A washing step to eliminate the excess of PEMAX was required, so supernatant was eliminated and 500 μl of sterile SCP buffer was added. Samples were collected under the same centrifugation conditions. Non-PEMAX treated samples, prepared with 20 μl of SCP buffer plus 180 μl of viable and dead cells, were also analysed. DNA extraction of all samples was carried out as described above and qPCR was performed according to the conditions described initially and using the primer pair XF16S forward and probe and its reverse 3 (XF16S-3). Signal reduction (SR), defined as the difference between cycle threshold values (Δ*C*_*T*_) of non-PEMAX treated and PEMAX treated samples, was calculated to determine the effect of PEMAX concentration on DNA amplification suppression by qPCR assay. Three biological replicates were performed.

### Evaluation of v-qPCR with Xff, Xfp and Xfm strains

The v-qPCR sensitivity and amplification efficiency was evaluated with standard curves. Suspensions of viable and dead Xff, Xfp and Xfm cells were prepared in SCP as described above. Samples were prepared to cover a 7-log range (from 1 × 10^2^ CFU/ml to 1 × 10^8^ CFU/ml) in Xff and a 6-log range (from 1 × 10^2^ CFU/ml and up to 1 × 10^7^ CFU/ml) in Xfp and Xfm. Mixture suspensions were also prepared, with the same concentration range of viable Xf cells in addition to a constant number of dead cells (1 × 10^6^ CFU/ml). From each suspension, 180 μl were treated with PEMAX at 7.5 μM according to the procedure described previously, and 180 μl were used as non-PEMAX treated sample. DNA extraction was performed as described in both PEMAX treated and non-PEMAX treated samples. qPCR was performed as described previously, each reaction per duplicate and using XF16S-3 as the primer pair. Standard curves were generated plotting *C*_*T*_ values obtained against the logarithm of the initial number of CFU/ml, and the amplification efficiency was calculated as described above.

### Evaluation of v-qPCR for antimicrobial activity assessment

The contact test conditions were optimized for the antimicrobial activity assessment of AMPs against Xf. Xff cell concentration, contact test time and peptide concentration were evaluated. The peptide **BP178** (Table 2) was used [19]. Lyophilized **BP178** was solubilized in sterile Milli-Q water to a final concentration of 1 mM, filter sterilized through a 0.22 μm pore filter and 10X stock solutions of the desired concentrations were prepared in sterile distilled water. Suspensions of Xff cells prepared in sterile SCP buffer were used, and 20 μl of each **BP178** stock concentration were mixed in 1.5 ml tubes with 160 μl of the corresponding Xff cell suspension and incubated for 1.5, 3, 6, 24, or 48 h depending on the experiment. After the incubation period, 20 μl of PEMAX or SCP buffer were added to the samples for v-qPCR or qPCR, respectively, before DNA extraction.

In a first experiment, suspensions of Xff cells at 1 × 10^7^ and 1 × 10^8^ CFU/ml were tested to determine the differences in log reduction when using **BP178** final concentrations of 1.6, 12.5 and 50 μM. A second experiment was used to evaluate different contact test times in order to select the most suitable one for the assays. Additionally, in a third experiment, **BP178** concentrations of 3.1, 6.2, 12.5, 25 and 50 μM were incubated with Xff at 1 × 10^7^ CFU/ml for 3 and 24 h to determine the most informative peptide concentrations to screen the peptides.

A non-treated control (Xff cells without peptide) using SCP buffer instead of peptide was also included in all the experiments, and three replicates for each Xff cell concentration, contact test time and peptide concentration were used. Xff log_10_ CFU/ml of the initial cell suspensions and of the contact tests, with or without the peptide, was determined using qPCR (total cells), v-qPCR (viable cells) and plate counting (culturable cells). For assessment of total, viable and culturable cells, aliquots were taken from the contact test wells at given times.

For qPCR and v-qPCR, DNA was isolated from two individual samples of 200 μl of each contact test, in the case of v-qPCR, previously the sample was treated with PEMAX at final concentration of 7.5 μM as described above. DNA extraction, qPCR analysis using the TaqMan-based qPCR assay XF16S-3 and quantification were performed as described above. The amount of total and viable cells was obtained by interpolating the *C*_*T*_ values from each sample against the respective standard curve and expressed as log_10_ CFU/ml. For plate counting, each sample was serially diluted, and appropriate dilutions were seeded onto PD2 agar plates. Plates were incubated at least for 1 week at 28 °C, colonies were counted and CFU/ml value was determined for each sample. The MBC of **BP178** was determined in order to compare with other described peptides. The MBC corresponds to the lowest concentration where no growth was detected in plate counting after exposure to the peptide in the contact test.

### Screening of peptide conjugates derived from BP100 against Xff

A set of peptide conjugates derived from peptide **BP100** reported by our group as AMPs (Table 2) [17] were selected to be screened against Xff. The epitope tag peptide **tag54** was used as a negative control and **BP100** and **BP178** was included for comparison purposes. All AMPs were evaluated as described above, at final concentrations of 3.1 and 12.5 μM and against a suspension of Xff at 1 × 10^7^ CFU/ml using a 3 h contact test. After the incubation period, Xff population level was assessed using v-qPCR as described above. Loss of viability after the contact test was calculated and expressed as logarithmic reduction of Xff population. Three replicates for each AMP and concentration were used. After the screening, **BP171** and **BP198**, showing different antibacterial activity against Xff, were selected to assess the performance of the v-qPCR methodology for the quantification of the viable Xff population. Peptide concentrations of 1.5, 3.1, 6.2, 12.5 and 25 μM were incubated with Xff at 1 × 10^7^ CFU/ml for 3 h to determine viable and culturable cells by v-qPCR and plate counting respectively. Three replicates for each AMP and concentration were used.

### Statistical analysis

To test the significance of the effect of PEMAX concentration in the suppression of DNA amplification (signal reduction) on dead and viable cells of Xff, a one-way analysis of variance (ANOVA) was performed. To test the significance of the parameters studied to set up the conditions of the contact test (initial Xff cell concentration, contact test time and peptide concentration) and of the cell quantification method, a two or three-way ANOVA were performed. To test the effect of AMPs on Xff viability reduction a one-way ANOVA was performed. In all cases, means were separated according to the Tukey’s test at a *P* value of ≤0.05.

## Supplementary information

**Additional file 1.** Standard curves of the eight qPCR assays studied. Each set of primer pairs amplifying the same target gene with different amplicon lengths are shown in the same box, (A) 16S rRNA gene (XF16S), (B) EFTu gene (EFTu), and (C) conserved hypothetical protein (HL). The equations of the curves are shown for each primer pair.

**Additional file 2. **Signal reduction (SR) in the qPCR of viable (white) and dead (grey) cells after treatment with different PEMAX concentrations. SR is the difference between the *C*_*T*_ value of non-PEMAX and PEMAX treated cells. Cell concentration was 1 × 10^7^ CFU/ml. TaqMan-based qPCR assay XF16S-3 (amplicon length of 279 bp) was used for this experiment. The results are shown as the mean from three independent replicates, and error bars represent standard deviation of the means. Lowercase letters correspond to the means comparison of SR in viable cells. Capital letters correspond to the means comparison of SR in dead cells. Means sharing the same letters are not significantly different (*P* < 0.05), according to the Tukey’s test.

**Additional file 3. **Effect of peptides **BP171** (circles) and **BP198** (triangles) on viability and culturability of Xff strain Temecula at different peptide concentrations. Cell viability was estimated by v-qPCR (black symbols), and cell culturability by plate counting (grey symbols). An exposure time of 3 h and a cell concentration of 1 × 10^7^ CFU/ml were used in both cases. The dash line represents the detection limit of v-qPCR, whereas the normal line indicates the detection limit of the plate counting technic. Values are the means of three replicates, and error bars represent the standard deviation of the mean.

## Data Availability

The datasets used and/or analysed during the current study are available from the corresponding author on reasonable request.

## Abbreviations

Xf: *Xylella fastidiosa*
AMPs: Antimicrobial peptides
VBNC: Viable-but-non-culturable
CTC: 5-cyano-2,3-ditolyl tetrazolium chloride
qPCR: Quantitative PCR
v-qPCR: Viable quantitative PCR
PMA or PMAxx: Propidium monoazide
EMA: Ethidium monoazide
CFU: Colony forming units
*C*_*T*_: Cycle threshold
Xff: *Xylella fastidiosa* subsp. *fastidiosa*
Xfp: *Xylella fastidiosa* subsp. *pauca*
Xfm: *Xylella fastidiosa* subsp. *multiplex*
SR: Signal reduction
Δ*C*_*T*_: Difference between cycle threshold values
MBC: Minimal bactericidal concentration
MIC: Minimal inhibitory concentration
PD2: Pierce Disease 2
BCYE: Buffered charcoal yeast extract
SCP: Succinate-citrate-phosphate
ANOVA: Analysis of variance
