# Supplementary material for: Screening and identification of BP100 peptide conjugates active against Xylella fastidiosa using a viability-qPCR method
Source: BMC Microbiol. 2020 Jul 29;20:229. doi: 10.1186/s12866-020-01915-3 (PMC7392676; doi:10.1186/s12866-020-01915-3)
Supplement: Supplementary file 1 — Additional file 1. Standard curves of the eight qPCR assays studied. Each set of primer pairs amplifying the same target gene with different amplicon lengths are shown in the same box, (A) 16S rRNA gene (XF16S), (B) EFTu gene (EFTu), and (C) conserved hypothetical protein (HL). The equations of the curves are shown for each primer pair. [file 12866_2020_1915_MOESM1_ESM.pdf]

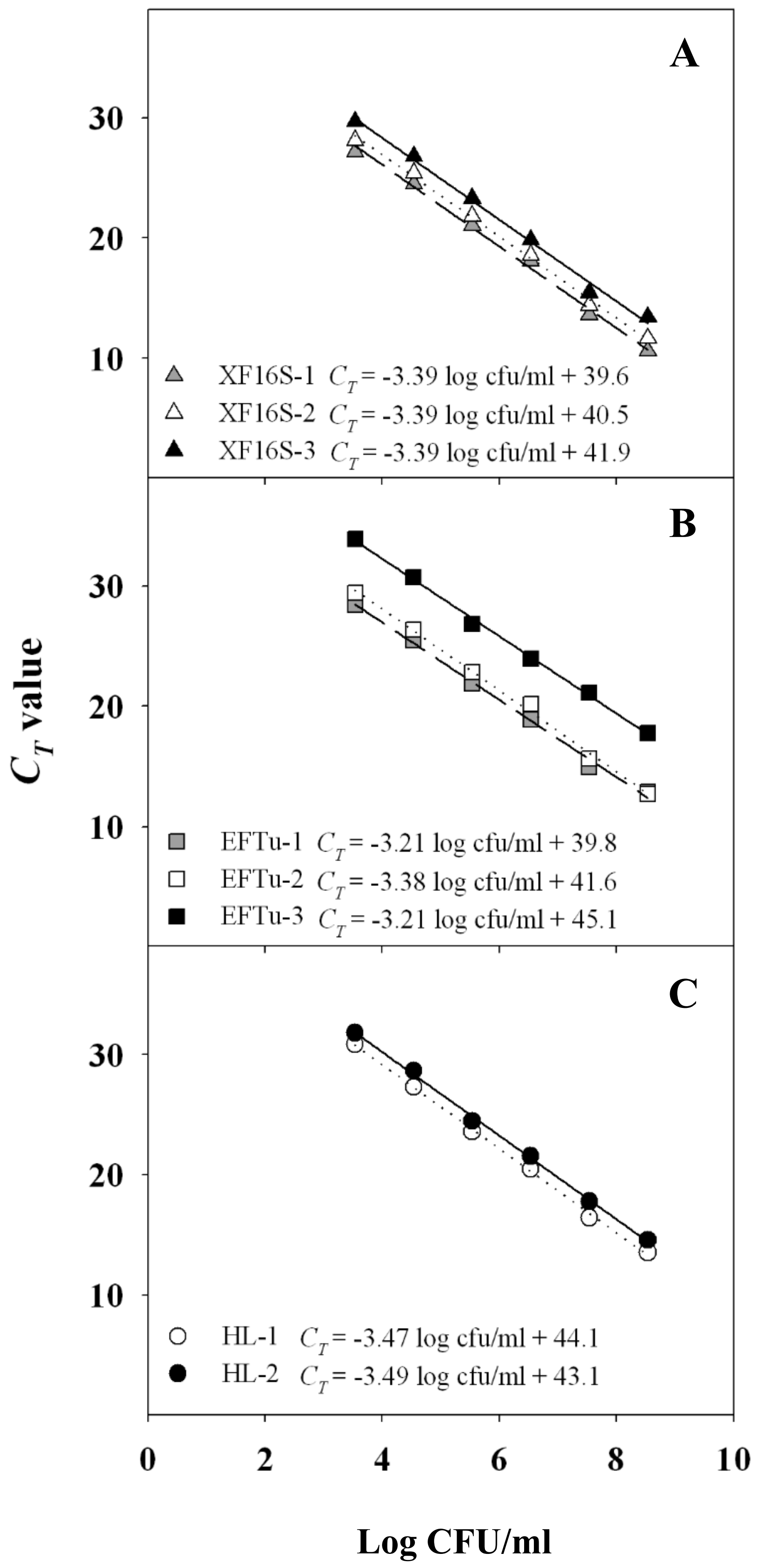

Additional file 1. Standard curves of the eight qPCR assays studied. Each set of primer pairs amplifying the same target gene with different amplicon lengths are shown in the same box, (A) 16S rRNA gene (XF16S), (B) EFTu gene (EFTu), and (C) conserved hypothetical protein (HL). The equations of the curves are shown for each primer pair.
