# Supplementary material for: Screening and identification of BP100 peptide conjugates active against Xylella fastidiosa using a viability-qPCR method
Source: BMC Microbiol. 2020 Jul 29;20:229. doi: 10.1186/s12866-020-01915-3 (PMC7392676; doi:10.1186/s12866-020-01915-3)
Supplement: Supplementary file 2 — Additional file 2. Signal reduction (SR) in the qPCR of viable (white) and dead (grey) cells after treatment with different PEMAX concentrations. SR is the difference between the CT value of non-PEMAX and PEMAX treated cells. Cell concentration was 1 × 10^7^ CFU/ml. TaqMan-based qPCR assay XF16S-3 (amplicon length of 279 bp) was used for this experiment. The results are shown as the mean from three independent replicates, and error bars represent standard deviation of the means. Lowercase letters correspond to the means comparison of SR in viable cells. Capital letters correspond to the means comparison of SR in dead cells. Means sharing the same letters are not significantly different (P < 0.05), according to the Tukey’s test. [file 12866_2020_1915_MOESM2_ESM.pdf]

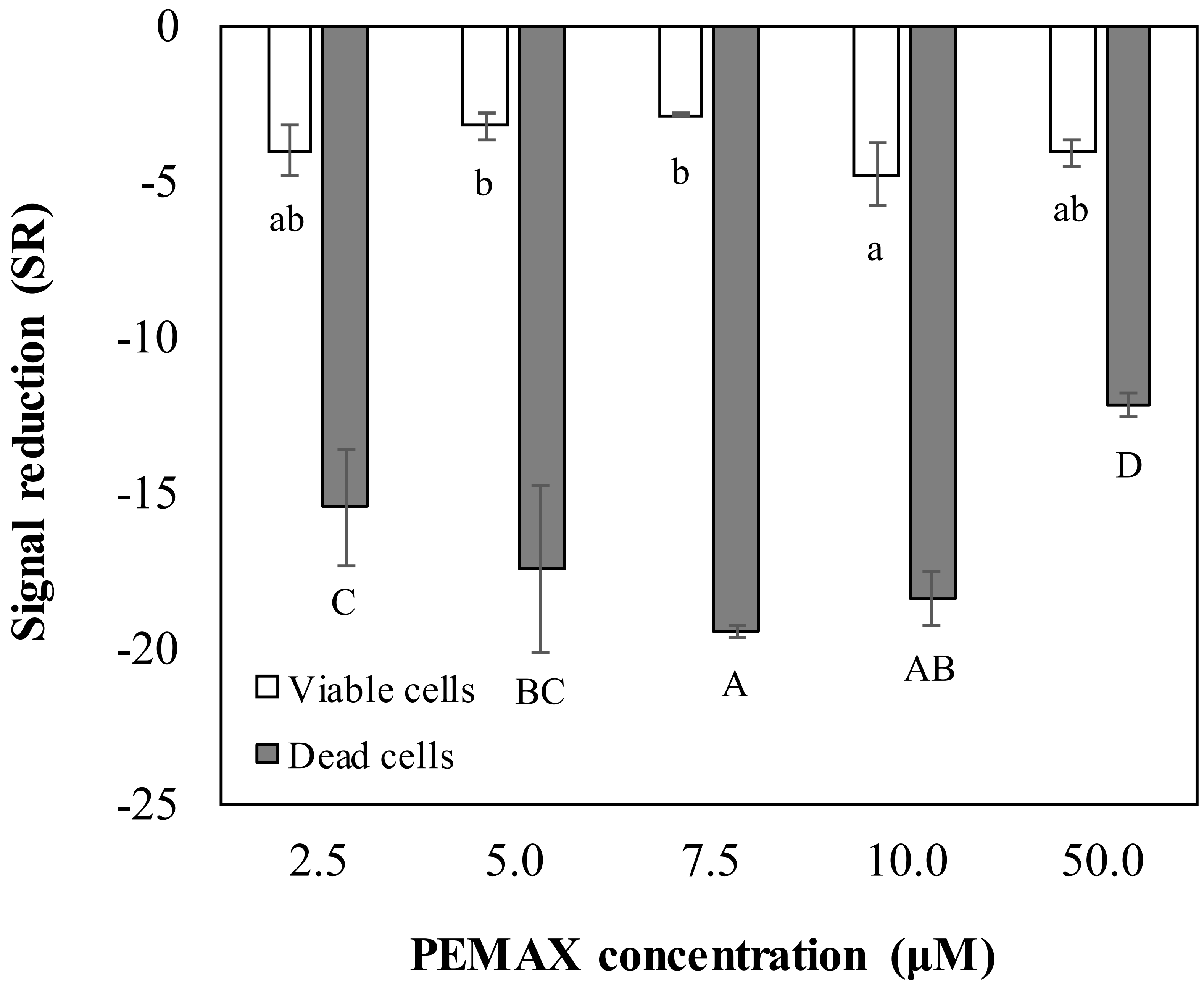

Additional file 2. Signal reduction (SR) in the qPCR of viable (white) and dead (grey) cells after treatment with different PEMAX concentrations. SR is the difference between the C_T value of non-PEMAX and PEMAX treated cells. Cell concentration was 1 x 10^7 CFU/ml. TaqMan-based qPCR assay XF16S-3 (amplicon length of 279 bp) was used for this experiment. The results are shown as the mean from three independent replicates, and error bars represent standard deviation of the means. Lowercase letters correspond to the means comparison of SR in viable cells. Capital letters correspond to the means comparison of SR in dead cells. Means sharing the same letters are not significantly different ($P < 0.05$), according to the Tukey's test.
