# Supplementary material for: Screening and identification of BP100 peptide conjugates active against Xylella fastidiosa using a viability-qPCR method
Source: BMC Microbiol. 2020 Jul 29;20:229. doi: 10.1186/s12866-020-01915-3 (PMC7392676; doi:10.1186/s12866-020-01915-3)
Supplement: Supplementary file 3 — Additional file 3. Effect of peptides BP171 (circles) and BP198 (triangles) on viability and culturability of Xff strain Temecula at different peptide concentrations. Cell viability was estimated by v-qPCR (black symbols), and cell culturability by plate counting (grey symbols). An exposure time of 3 h and a cell concentration of 1 × 10^7^ CFU/ml were used in both cases. The dash line represents the detection limit of v-qPCR, whereas the normal line indicates the detection limit of the plate counting technic. Values are the means of three replicates, and error bars represent the standard deviation of the mean. [file 12866_2020_1915_MOESM3_ESM.pdf]

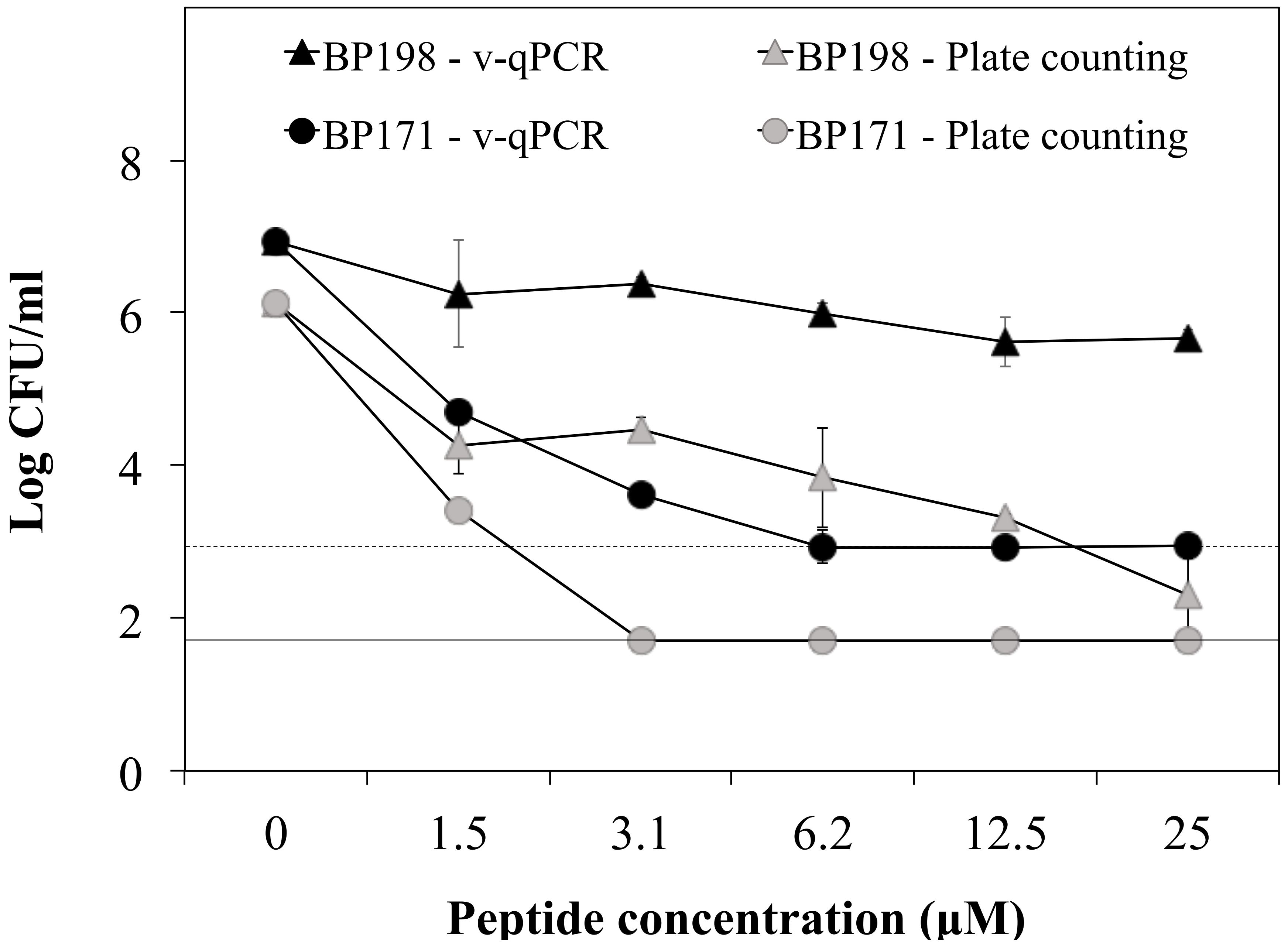

Additional file 3. Effect of peptides **BP171** (circles) and **BP198** (triangles) on viability and culturability of Xff strain Temecula at different peptide concentrations. Cell viability was estimated by v-qPCR (black symbols), and cell culturability by plate counting (grey symbols). An exposure time of 3 h and a cell concentration of 1 x 10^7 CFU/ml were used in both cases. The dash line represents the detection limit of v-qPCR, whereas the normal line indicates the detection limit of the plate counting technic. Values are the means of three replicates, and error bars represent the standard deviation of the mean.
